# SARS-CoV-2 failure to infect or replicate in mosquitoes: an extreme challenge

**DOI:** 10.1038/s41598-020-68882-7

**Published:** 2020-07-17

**Authors:** Yan-Jang S. Huang, Dana L. Vanlandingham, Ashley N. Bilyeu, Haelea M. Sharp, Susan M. Hettenbach, Stephen Higgs

**Affiliations:** 1 Department of Diagnostic Medicine and Pathobiology, College of Veterinary Medicine, Kansas State University, Manhattan, KS 66506 USA; 2 Biosecurity Research Institute, Kansas State University, Manhattan, KS 66506 USA

**Keywords:** Ecology, Diseases

## Abstract

This research addresses public speculation that SARS-CoV-2 might be transmitted by mosquitoes. The World Health Organization has stated “To date there has been no information nor evidence to suggest that the new coronavirus could be transmitted by mosquitoes”. Here we provide the first experimental data to investigate the capacity of SARS-CoV-2 to infect and be transmitted by mosquitoes. Three widely distributed species of mosquito; *Aedes aegypti*, *Ae. albopictus* and *Culex quinquefasciatus*, representing the two most significant genera of arbovirus vectors that infect people, were tested. We demonstrate that even under extreme conditions, SARS-CoV-2 virus is unable to replicate in these mosquitoes and therefore cannot be transmitted to people even in the unlikely event that a mosquito fed upon a viremic host.

## Introduction

The question has been asked as to whether or not SARS-CoV-2, the causative agent of COVID-19, can infect and be transmitted by mosquitoes. The WHO has definitively stated that mosquitoes cannot transmit the virus^1^, and in interviews, various experts have unanimously and definitively also dispelled the suggestion that SARS-CoV-2 could be transmitted by mosquitoes. The presumption may be based on various observations and facts extrapolated from other coronaviruses. For example, neither the closely related SARS-CoV nor MERS produce the level of virus in the blood that for typical arthropod-borne viruses such as dengue and yellow fever, would be regarded as high enough to infect mosquitoes. Recent studies with infected humans and non-human primates infected with SARS-CoV-2, found no detectable virus in peripheral blood^2,3^. Lack of viremia is also suggested by the fact that neither SARS-CoV nor MERS infections have resulted from blood transfusions or organ transplantations. Since mechanical transmission of viruses by arthropods requires a very high viremia^4^, even if mosquitoes were interrupted when feeding on a SARS-CoV-2 infected person, the mouthparts would not be contaminated. Although we do not know the duration of virus infectivity on contaminated surfaces, mechanical transmission by non-hematophagous arthropods seems highly unlikely, and even if not impossible, would result in very few, if any human infections, and not be epidemiologically relevant. Despite the lack of detectable viremia, experiments to determine the potential role of mosquitoes in SARS-CoV-2 transmission, are necessary because previous experiments have demonstrated that mosquitoes may become infected with viruses even when exposed to levels of infectious virus that are below the level of detection^5–7^.

To be a biological vector of viruses, mosquitoes must take up sufficient virus to infect midgut epithelial cells, and the virus must then disseminate to infect other organs in the hemocoel, notably the salivary glands. Overcoming the midgut infection and escape barriers is essential for a virus to be transmissible by mosquitoes. If these barriers are bypassed by direct inoculation of virus into the hemocoel, then even non-susceptible mosquitoes can be infected. Intrathoracic inoculation^8,9^ of virus directly into the hemocoel can accomplish short-term infection of insects that could never be naturally infected because they do not feed on blood. These include not only non-hematophagous mosquitoes such as *Toxorhynchites *spp, but also male mosquitoes and even beetles and butterflies^10,11^. The use of intrathoracic inoculation, also addressed published reports that the natural physical breaching of the midgut wall by filarial, may enable a disseminated coinfection of viruses in resistant mosquitoes^12^.

Similar to over 500 viruses that are transmitted by arthropods^13^, with the exception of African swine fever virus, coronaviruses have an RNA genome. In spite of the recovery of coronavirus or coronavirus-like agents from various arthropods^14,15^, no virus in the family has been isolated from mosquitoes. To date, only one report related to epidemic coronaviruses and mosquitoes has been published^16^. This study that evaluated the potential use of mosquitoes for surveillance, included feeding of MERS virus to *Anopheles gambiae* mosquitoes. Residual viral RNA, probably in the remains of the bloodmeal in the midgut, was detected up to 1-day post-feeding. Similarly, positive PCR detection was observed for *Bacillus anthracis*, *Trypanosoma brucei gambiensis,* and Zika virus, none of which infect or are transmitted by *An. gambiae*. Levels of detected RNA were equal to or below the input level, indicating a lack of replication. By analyzing samples using in vitro cultivation, rather than using molecular approaches, we focused specifically on detection of infectious virus rather than on RNA. As illustrated by, for example, the use of inactivation techniques specifically developed to enable safe handling and shipping of viral material, the mere presence of RNA does not mean that any infectious virus is actually present. It is well known that viral RNA can be detected in mosquitoes simply because they have fed on a viremic host, and so RNA detection should never be interpreted as proof of mosquito susceptibility to infection and competence to transmit the virus.

## Results

In this study, the susceptibility of three mosquito species, *Ae. aegypti*, *Ae. albopictus* and *Cx. quinquefasciatus*, were determined through the intrathoracic inoculation with SARS-CoV-2. Infectious viruses were recovered from 13/15 mosquitoes collected within two hours of inoculation. It is possible, that in the two negative mosquitoes, the inoculated virus lost infectivity during the holding period. No virus was detected in the 277 inoculated mosquitoes collected and titrated at time points beyond 24 h, suggesting a rapid loss of infectivity and the lack of replication after injection. From a total of 48 mosquitoes analyzed, infectious viruses were only recovered from one *Ae. albopictus* collected at 24 h post-inoculation. The quantity of infectious virus in this mosquito corresponded to the amount of inocula, producing infectious titers at approximately 1.5 logTCID_50_/ml. No virus was detected in control L-15 medium inoculated mosquitoes. Collectively, our findings suggest that mosquitoes in the *Aedes* and *Culex* genera are refractory to SARS-CoV-2 and unlikely to contribute to viral maintenance and transmission in nature (Table 1.).

**Table 1:** Recovery rates of SARS-CoV-2 in mosquitoes receiving intrathoracic injection.

Species	Inoculum	Time (days post infection)					
		0*	1	3	7	10	14
*Ae. aegypti*	Mock**	0.0% (0/6)	0.0% (0/3)	0.0% (0/5)	0.0% (0/5)	0.0% (0/6)	0.0% (0/5)
	SARS-CoV-2	83.3% (5/6)	0.0% (0/17)	0.0% (0/17)	0.0% (0/24)	0.0% (0/26)	0.0% (0/27)
*Ae. albopictus*	Mock**	0.0% (0/6)	0.0% (0/4)	0.0% (0/2)	0.0% (0/4)	0.0% (0/2)	0.0% (0/5)
	SARS-CoV-2	83.3% (5/6)	7.1% (1/14)	0.0% (0/15)	0.0% (0/20)	0.0% (0/21)	0.0% (0/32)
*Cx. quinquefasciatus*	Mock**	0.0% (0/3)	0.0% (0/1)	N/A	0.0% (0/1)	N/A	0.0% (0/1)
	SARS-CoV-2	100.0% (3/3)	0.0% (0/17)	0.0% (0/17)	0.0% (0/25)	0.0% (0/28)	0.0% (0/25)

* Mosquitoes obtained at day 0 post infection were collected within 2 h from the time of intrathoracic injection.

** All mock control groups of mosquitoes received Leibovitz’s L-15 media.

## Discussion

The most extreme approach for viral challenge of mosquitoes, namely intrathoracic inoculation, was used as an ultimate test of the capacity of SARS-CoV-2 to infect and replicate in mosquitoes. The hypothesis was that if the virus did not replicate in mosquitoes after intrathoracic inoculation, then even if mosquitoes did feed on viremic people, and the virus disseminated from the midgut, the lack of replication would preclude the possibility of biological transmission. Three widely distributed species of mosquito, representing the two most significant genera of arbovirus vectors that infect people, were tested. All three of the species: *Aedes aegypti*, *Ae. albopictus,* and *Culex quinquefasciatus* are present in China, the country of origin of SARS-CoV-2. Samples collected within two hours of inoculation confirmed efficient delivery of infectious viruses to mosquitoes. Based upon the lack of detectable infectious virus in any of the 277 samples collected at all time points beyond 24 h post-inoculation, we conclude that SARS-CoV-2 is unable to replicate in mosquitoes and that even if a mosquito fed on a person with virus in the blood, that the mosquito would not be a vector if feeding on a naïve host.

## Methods

Virus: SARS-CoV-2 virus WA1/2020 strain was obtained from BEI Resources (Catalog # NR-52281). Virus was propagated in Vero76 cells at the approximate multiplicity of infection of 0.01. Using serial tenfold dilutions in 96-well plates^17^, infectious titers of viral stocks used for intrathoracic injection were approximately 5.5 logTCID_50_/ml.

Mosquitoes: The colonized *Aedes aegypti* strain Rex D, Higgs white eye was originally obtained from Puerto Rico^18^, *Ae. albopictus* generation F11 originated from New Jersey, and *Culex quinquefasciatu*s F43 were from Florida^19,20^. All mosquitoes were reared at 28 °C, relative humidity of 80% and a 12 h light:12 h dark photoperiod. These colonized mosquitoes have proven to be susceptible to several arboviruses^19,21–26^.

Viral challenge of mosquitoes: For intrathoracic inoculation^9^, mosquitoes were cold-anaesthetized on ice, transferred to a secure glove box, and then inoculated with approximately 0.5 µl of viral stock. It was anticipated that each mosquito received approximately 2.0 logTCID_50_/ml of infectious viruses. L-15 medium was inoculated as a negative control. The results were compiled from two experiments using *Ae. aegypti* and *Ae. albopictus* and one experiment using *Cx. quinquefasciatus*. Experimentally challenged mosquitoes were maintained and sampled under conditions as described above. Mosquitoes were individually triturated in 1 ml of medium using a TissueLyser II platform (Qiagen, Valencia, CA), and titrated on Vero cells as previously described.
